# Estimating the False Discovery Rate Using Mixed Normal Distribution for Identifying Differentially Expressed Genes in Microarray Data Analysis

**Published:** 2008-01-22

**Authors:** Akihiro Hirakawa, Yasunori Sato, Takashi Sozu, Chikuma Hamada, Isao Yoshimura

**Affiliations:** 1 Genetics Division, National Cancer Center Research Institute, Chuo-ku, Tokyo, Japan; 2 The Center for Advanced Medical Engineering and Informatics, Osaka University, Suita, Osaka, Japan; 3 Faculty of Engineering, Tokyo University of Science, Shinjuku-ku, Tokyo, Japan

**Keywords:** differentially expressed genes, false discovery rate, microarray, mixed normal distribution, significance analysis of microarray

## Abstract

The recent development of DNA microarray technology allows us to measure simultaneously the expression levels of thousands of genes and to identify truly correlated genes with anticancer drug response (differentially expressed genes) from many candidate genes. Significance Analysis of Microarray (SAM) is often used to estimate the false discovery rate (FDR), which is an index for optimizing the identifiability of differentially expressed genes, while the accuracy of the estimated FDR by SAM is not necessarily confirmed. We propose a new method for estimating the FDR assuming a mixed normal distribution on the test statistic and examine the performance of the proposed method and SAM using simulated data. The simulation results indicate that the accuracy of the estimated FDR by the proposed method and SAM, varied depending on the experimental conditions. We applied both methods to actual data comprised of expression levels of 12,625 genes of 10 responders and 14 non-responders to docetaxel for breast cancer. The proposed method identified 280 differentially expressed genes correlated with docetaxel response using a cut-off value for achieving FDR <0.01 to prevent false-positive genes, although 92 genes were previously thought to be correlated with docetaxel response ones.

## Introduction

Genetic markers are promising for our ability to predict the anticancer drug response in individual patients. The recent development of DNA microarray technology allows us to measure simultaneously the expression levels of thousands of genes and to identify truly correlated genes with the anticancer drug response, called differentially expressed genes, from many candidate genes by comparing the gene expression levels between cells or tissues under different conditions. However, since a typical microarray experiment measures the expression levels of thousands of genes with a small sample-size simultaneously, identifying differentially expressed genes poses complex multiple testing problems, and it is difficult to precisely identify differentially expressed genes using traditional statistical methods. The traditional methods such as the two-sample *t*-test have been used to identify differentially expressed genes [15]. However, such tests often provide unreliable and inaccurate results due to strong parametric assumptions and multiple testing problems. In contrast, Bonferroni correction [5] controlling the family-wise error rate (FWER) is often too conservative, failing to identify differentially expressed genes. In order to solve this problem, the false discovery rate (FDR) is increasingly used. The FDR is defined as the expected proportion of false-positive genes among total identified genes as an index for optimizing the identifiability of differentially expressed genes [4]. Many statistical methods have been proposed for estimating the FDR, i.e. empirical Bayes (EB) method [12], Significance Analysis of Microarray (SAM) [34], and mixture model method (MMM) [24]. Among them, SAM is most widely used for cancer outcome by its attractive advantages in microarray data analysis [10]. Actually, the difficulty of multiplicity problems in simultaneous testing of a large number of genes with a small sample-size data is relieved by SAM through estimating the number of false-positive genes based on a permutation procedure without strict parametric assumptions and replacing the usual *t*-test statistic with a SAM-statistic [34] or *t*-type score [24]. Available computer software specific for SAM, also help biological researchers for managing SAM [9]. The precision of estimated FDR in SAM have been examined by many researchers [14, 22, 23, 36]. Among them, Xie et al. (2005) pointed out that the permutation-based methods for FDR estimation such as SAM might overestimate FDR in a certain condition. This suggests the importance of the examination of factors such as target FDR, sample-size, and proportion of differentially expressed genes which may affect the bias and variance of estimated FDR in SAM. If the bias and variance of estimated FDR differ, depending on the experimental condition, we have to choose a suitable method for the experimental condition in a confronted case. We therefore, conducted a simulation study to examine the bias and variance of estimated FDR in SAM.

In this paper, we also propose a new method for estimating the FDR. The proposed method assumes a mixed normal distribution on *t*-type score, estimating the FDR for a cut-off value based on the numerical integration of probability distribution. Here, the *t*-type score is a test statistic with a correction term added to the denominator of the Welch type *t*-statistic in order to stabilize the variation of the denominator [24]. We compared both bias and variance of the estimated FDR between the proposed method and SAM through the simulation study. Additionally, both methods are applied to actual data comprised of the expression levels of 12,625 genes of 10 responders and 14 non-responders to docetaxel for breast cancer (Accession No: GDS360) [20]. Although 92 correlated genes with the docetaxel response were previously identified using a two-sample *t*-test with the significance level 0.001 [7], there are many false-negative genes among unidentified genes because the adopted significance level is too low to get reasonable result. We, therefore, examined the FDR in this actual data using the proposed method.

## Materials and Methods

### *t*-type score

For each gene *i*, *i =* 1, 2, …, *g*, the expression level is *X_i_*_1_, …, *X_im_* from *m* samples collected from cells or tissues under Condition 1, and *Y_i_*_1_, …, *Y_in_* from *n* samples collected from cells or tissues under Condition 2. A traditional method for testing for a difference in the means between two conditions assuming a normal distribution is the two-sample *t*-test. However, since thousands of genes are evaluated simultaneously; when some of them have a very small sum of squares under two conditions, their absolute *t*-statistic becomes very large even though their mean difference is not large. This disadvantage is exacerbated due to the small sample-size. In the case where two-sample *t*-test is used, therefore, many non-differentially expressed genes are identified as differentially expressed genes. In order to avoid this problem, a new statistic with a correction term added to the denominator of the Welch type *t*-statistic in order to stabilize the variation of its denominator, called *t*-type score, has been proposed [24]. We use the *t*-type score as a test statistic for identifying the differentially expressed genes. Let *z_i_* denote the *t*-type score for gene *i*,

(1) $$z_{i}=\frac{{\bar{X}}_{i}-{\bar{Y}}_{i}}{\sqrt{s_{Xi}^{2}/m+s_{Yi}^{2}/n}+a_{0}}$$

where ${\bar{X}}_{i}=\sum_{j=1}^{m}X_{ij}/m$ and ${\bar{Y}}_{i}=\sum_{j=1}^{n}Y_{ij}/n$ are the sample means for gene *i* under two conditions respectively, and $S_{Xi}^{2}=\sum_{j=1}^{m}{\left(X_{ij}-{\bar{X}}_{i}\right)}^{2}/(m-1)$ and $S_{Yi}^{2}=\sum_{j=1}^{n}{\left(Y_{ij}-{\bar{Y}}_{i}\right)}^{2}/(n-1)$ are the sample variances for gene *i*, *a*_0_ is the 90th percentile of $\left\{\sqrt{S_{Xi}^{2}/m+S_{Yi}^{2}/n:}i=1,\ldots ,g\right\}$.

### Significance analysis of microarray (SAM)

SAM is often used to estimate the FDR for identifying the differentially expressed genes for cancer outcome [10]. The FDR is estimated through the replications of permutation among all samples for a total of *B* times. For the *b*th permutated data, the *t*-type score is calculated and denoted by $z_{i}^{b}$ *i =* 1, …, *g*. When *FDRsam* denotes the two-sided FDR estimator, *FDRsam* can be written as

(2) $$F\hat{D}Rsam=\frac{\frac{1}{B}\sum_{b=1}^{B}\#\{i|z_{i}^{b}\geq c_{1}\cup z_{i}^{b}\leq c_{2}\}}{\#\{i|z_{i}\geq c_{1}\cup z_{i}\leq c_{2}\}},$$

where *c*_1_ (>0) and *c*_2_ (<0) are the cut-off values, respectively. We can identify over- and under-expressed genes simultaneously using the *FDRsam*. On the other hand, we formulate the one-sided FDR estimator for each cut-off value (*c*_1_, *c*_2_) in order to correspond to the FDR estimator of the proposed method. When *FDRsam*(*c*_1_) and *FDRsam*(*c*_2_) denote the one-sided FDR estimator for *c*_1_ and *c*_2_ respectively, *FDRsam*(*c*_1_) and *FDRsam*(*c*_2_) can be written as

(3) $$F\hat{D}Rsam(c_{1})=\frac{\frac{1}{B}\sum_{b=1}^{B}\#\{i|z_{i}^{b}\geq c_{1}\}}{\#\{i|z_{i}\geq c_{1}\}}$$

and

(4) $$F\hat{D}Rsam(c_{2})=\frac{\frac{1}{B}\sum_{b=1}^{B}\#\{i|z_{i}^{b}\leq c_{2}\}}{\#\{i|z_{i}\leq c_{2}\}},$$

respectively.

The *FDRsam*(*c*_1_) is used in order to identify the differentially expressed genes that the gene expression levels under Condition 1 over-express more than under Condition 2. On the other hand, the *FDRsam*(*c*_2_) is used in order to identify the differentially expressed genes that the gene expression levels under Condition 1 under-express more so than under Condition 2.

### Proposed FDR estimation method

We propose estimating the FDR assuming a *K*-component mixed normal distribution on *t*-type score *z_i_*, *i* = 1, ..., *g*. The probability density function of *K*-component mixed normal distribution is

(5) $$f(z;\theta )=\sum_{k=1}^{K}p_{k}f_{k}(z;\Delta_{k},V_{k}),$$

where *f_k_* (*z; Δ_k_*, *V_k_*) denotes the density function of a normal distribution *Normal* (*Δ_k_*, *V_k_*) with mean *Δ_k_*, and variance *V_k_*, and mixed proportion *p_k_*. *θ* represents all unknown parameters {*p_k_*, *Δ_k_*, *V_k_* : *k =* 1, ..., *K*} in a *K*-component mixed normal model. To estimate the all unknown parameters, given *z*_1_, …, *z_g_*, the following log-likelihood function is maximized.

(6) $$\text{log}\;\;L(\theta ;z)=\sum_{i=1}^{g}\text{log}\;\;f(z_{i};\theta )$$

To obtain the maximum likelihood estimat *θ̂*, the Newton-Raphson method is used. The one-sided FDR for each cut-off value (*c*_1_, *c*_2_) is estimated using the parameter estimates *θ̂*. When *P_TP_*_1_ and *P_TP_*_2_ denote the proportion of total identified positive genes for each cut-off value (*c*_1_, *c*_2_) respectively, *P_TP_*_1_ and *P_TP_*_2_ can be written as

(7) $${\hat{P}}_{TP1}=\int_{c_{1}}^{+\infty }f(z;\hat{\theta })\;\;dz,$$

and

(8) $${\hat{P}}_{TP2}=\int_{-\infty }^{c_{2}}f(z;\hat{\theta })\;\;dz,$$

respectively.

Let *P_FP_*_1_ and *P_FP_*_2_ denote the proportion of false-positive genes for each cut-off value (*c*_1_, *c*_2_) respectively, *P_FP_*_1_ and *P_FP_*_2_ can be written as

(9) $${\hat{P}}_{FP1}=\int_{c_{1}}^{+\infty }{\hat{p}}_{0}\;\;f_{0}(z;{\hat{\Delta }}_{0},{\hat{V}}_{0})\;\;dz,$$

(10) $${\hat{P}}_{FP2}=\int_{-\infty }^{c_{2}}{\hat{p}}_{0}\;\;f_{0}(z;{\hat{\Delta }}_{0},{\hat{V}}_{0})\;\;dz.$$

Note that *f*_0_(*z; Δ*_0_, *V*_0_) denotes the normal distribution with the smallest absolute mean among *f*_1_(*z; Δ*_1_, *V*_1_), …, *f_K_*(*z; Δ_K_*, *V_K_*), *Δ*_0_ = min (|*Δ*_1_|, …, |*Δ_K_*|). When *FDRp*(*c*_1_) and *FDRp*(*c*_2_) denote the one-sided FDR estimator for each cut-off value (*c*_1_, *c*_2_) respectively, *FDRp*(*c*_1_) and *FDRp*(*c*_2_) can be written as

(11) $$F\hat{D}Rp(c_{1})=\frac{\int_{c_{1}}^{+\infty }{\hat{p}}_{0}\;\;f_{0}(z;\;\;{\hat{\Delta }}_{0},{\hat{V}}_{0})dz}{\int_{c_{1}}^{+\infty }f(z;\hat{\theta })\;\;dz},$$

and

(12) $$F\hat{D}Rp(c_{2})=\frac{\int_{-\infty }^{c_{2}}{\hat{p}}_{0}\;\;f_{0}(z;\;\;{\hat{\Delta }}_{0},{\hat{V}}_{0})dz}{\int_{-\infty }^{c_{2}}f(z;\hat{\theta })\;\;dz},$$

respectively.

We can determine the cut-off value for the target one-sided FDR by changing *c*_1_ and *c*_2_ sequentially using Formula (11) and Formula (12).

### Simulation study to examine the performance of the proposed method and SAM

In usual microarray experiments, we evaluate the gene expression levels of thousands of genes simultaneously under various experimental conditions. Specifically, target FDR for determining the cut-off value, the sample-size, and the proportion of differentially expressed genes are varied depending on the experimental conditions. We therefore, examined the bias and variance of estimated FDR in both the proposed method and SAM under various experimental conditions through a simulation study. Although we conducted simulation experiments using a three-component model with over-expressed genes and under-expressed genes as well as a two-component model, this paper discusses the result obtained using the two-component model because the results of them were similar.

As the framework of simulation, we set the following simulation conditions.

#### Simulation condition 1

The simulation study was designed to have *g* (*i =* 1, …, *g*) genes in total, with *s* differentially expressed and *g*-*s* non-differentially expressed. Each condition had an equal sample-size *N* (*N* = *m = n*). We generated, for *j =* 1, …, *N*,

$$X_{ij}\;∼\;Normal\;(\mu_{i},\;0.5^{2}),\;i\;=\;1,\;...,\;s,$$

$$X_{ij}\;∼\;Normal\;(0.0,\;0.5^{2}),\;i\;=\;s\;+\;1,\;...,\;g,$$

and

$$Y_{ij}\;∼\;Normal\;(0.0,\;0.5^{2}),\;i\;=\;1,\;...,\;g,$$

respectively.

Since each population mean of differentially expressed genes was different respectively, we assumed a random effect model, that is, *μ_i_* ~ *Normal* (1.0, 0.1^2^), *i =* 1, …, *s*.

#### Simulation condition 2

The total number of replication of permutation (*B*) was 400 times in SAM.

#### Simulation condition 3

The proposed method assumes a two-component mixed normal distribution on the *t*-type score, estimating the parameters (θ̂) by the Newton-Raphson method.

The procedure for conducting the simulation study was as follows:

Step 1. Generate *X_ij_* and *Y_ij_* (*i =* 1, …, *g*, *j* = 1, …, *N*) according to Simulation Condition 1, calculating the *t*-type score (*z_i_*) of *g* genes including the *s* differentially expressed genes and *g*-*s* non-differentially expressed genes.

Step 2. Determine a cut-off value (*c*_1_) for target FDR (*tFDR*) by changing the cut-off value sequentially.

Step 3. In SAM, calculate the *t*-type score ( $z_{i}^{b}$, *i =* 1, …, *g*, *b =* 1, …, 400) using 400 permutated data according to Simulation Condition 2. In the proposed method, estimate the parameters (*θ*) of two-component mixed normal distribution according to Simulation Condition 3.

Step 4. Estimate the FDR using Formula (3) in SAM and Formula (11) in the proposed method for a cut-off value (*c*_1_).

Step 5. Repeat Steps 1 – 4 1,000 times, calculating the average of the bias of the estimated FDR and the variance of the estimated FDR in both methods.

The three situations of the simulation study were as follows:

#### Simulation situation 1

Each value is set as *g =* 3,000, *s =* 150, and *N =* 20, calculating the bias and variance of the estimated FDR in both methods when target FDR is set as *tFDR =* 0.01, 0.05, 0.1, 0.2, and 0.5 respectively.

#### Simulation situation 2

Each value is set as *tFDR =* 0.1, *g =* 3,000, and *s* = 150, calculating the bias and variance of the estimated FDR in both methods when sample-size is set as *N =* 5, 10, 20, 40, and 80 respectively.

#### Simulation situation 3

Each value was set as *tFDR =* 0.1, *g =* 3,000, and *N =* 20, calculating the bias and variance of the estimated FDR in both methods when the number of differentially expressed genes of the total genes is set as *s =* 30, 75, 150, 300, and 600 respectively.

## Results

### Results of simulation study

The bias and variance of the estimated FDR by both methods under each simulation situation are shown in Table 1, Table 2, and Table 3 respectively. Table 1 suggests that the bias and variance increase as target FDR becomes high in SAM, whereas the bias and variance were almost constant regardless of the target FDR in the proposed method. Table 2 suggests that the bias increases as the sample-size becomes large in SAM, whereas the bias decreased in the proposed method. In both methods, the variance was almost constant regardless of the sample-size. Table 3 suggests that the absolute bias increases as the number of the differentially expressed genes becomes large in SAM, whereas the bias decreases in the proposed method. In both methods, the variance decreases as the number of differentially expressed genes becomes large. Additionally, when *tFDR =* 0.5 or *s =* 600 in SAM and *N =* 5 or 10 in the proposed method, the absolute bias is larger than 0.01. The variance is smaller than that of SAM under all situations in the proposed method, except for *N =* 5.

### Application to actual data

We applied the proposed method and SAM to actual data comprised of the expression levels of 12,625 genes of 10 responders and 14 non-responders to docetaxel for breast cancer (Accession No: GDS360) [20]. This actual data was measured and analyzed in order to identify the correlated genes with the docetaxel response for predicting anti-tumor activity of individual patients [7]. Although 92 correlated genes with the docetaxel response were previously identified using a two-sample *t*-test (significance level 0.001), it was expected that there would be many false-negative genes among the genes that were not identified because a very strict significance level was used. We identified the correlated genes with docetaxel response based on the FDR using the proposed method and SAM.

**Figure 1.**
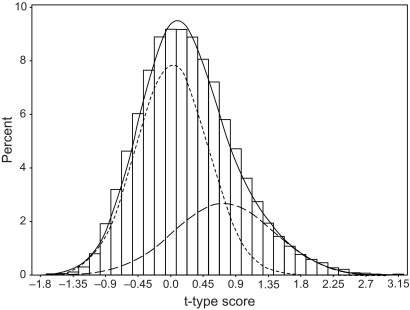
Histogram of the *t*-type score and the density function of a two-component mixed normal distribution. The solid line is *f*, the dotted line is *f*_0_, and the broken line is *f*_1_ in a two-component mixed normal distribution.

**Figure 2.**
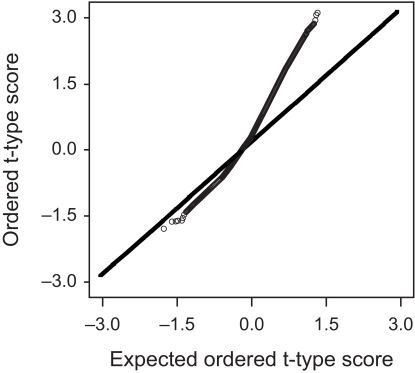
Scatter plot of the ordered *t*-type score versus the expected ordered *t*-type score in SAM.

In the proposed method, we assumed five mixed normal distributions on the *t*-type score with *K* = 2, …, 5, comparing their fitness by using Akaike Information Criterion (AIC) [1]. AIC is the most well-known criterion for determining the number of components in the model. As a result, we selected a two-component mixed normal distribution from the viewpoint of simplicity of interpretation, although AIC of the two-component model is almost equal to that of a three-component model. The density function of the two-component mixed normal distribution is *f*(*z*) = 0.319 *f*_1_(*z*; 0.659, 0.476) + 0.681 *f*_0_(*z*; − 0.057, 0.251). Figure 1 shows a histogram of the *t*-type score of 12,625 genes and the density function of a two-component mixed normal distribution. As shown in Figure 1, the two-component mixed normal distribution fits the *t*-type score well. We also calculated the order statistics of *z_i_*s from raw data, and the expected order statistics of $z_{i}^{b}$s from 1,000 permutated data. Figure 2 shows the scatter plot of the ordered *t*-type score versus the expected ordered *t*-type score in SAM. As shown in Figure 2, it is indicated that there are many differentially expressed genes.

## Discussion

While numerous research has been undertaken related to the bias of the estimated FDR by SAM [14, 22, 23, 36], little is known about the variance of the estimated FDR by SAM. Jung and Jang (2006) [14] noted that SAM can accurately estimate FDR when the target FDR is smaller than 0.1, which is an appropriate value in usual microarray data analysis. Pan (2002 Pan (2003) [22, 23] and Xie et al. (2005) [36] indicated the permutation-based methods for FDR estimation caused overestimation of FDR. In this paper, we examined both bias and variance of the estimated FDR by SAM under various experimental conditions through the simulation study in order to clarify the features of SAM. As a result of the simulation study, we uncovered some problems related to the SAM method. Estimating the distribution of non-differentially expressed genes using permutated data may not lead to precise estimation of FDR. Such a distribution based on the permutation is more dispersed than the true distribution of non-differentially expressed genes, resulting in overestimation of the number of false-positive genes. In particular, this disadvantage was influenced by the target FDR and the sample-size. In contrast, the proposed method estimates directly the distribution of non-differentially expressed genes assuming the mixed normal distribution on the *t*-type score. Although the estimated FDR by the proposed method was underestimated, the degree of bias of the estimated FDR in both the proposed method and SAM were almost same and the variance of the estimated FDR by the proposed method was smaller than that of SAM under all simulation situations, except for *N =* 5. The distribution based on the mixed normal distribution might be not more dispersed than the distribution based on the permutation. From the viewpoint of over-dispersion, therefore, the proposed method might precisely estimate the FDR than SAM.

In the simulation study, FDR tended to be underestimated in the proposed method and overestimated in SAM. Although the underestimation was not so large, this may cause the increase of false-positive genes. For instance, when 100 genes are identified as differentially expressed genes with the target FDR 0.1, truly false-positive genes are only 10 with the unbiased FDR, whereas more than 10 false-positive genes may be included in 100 by the underestimation of the FDR. To the contrary, the overestimation may cause the decrease of true-positive genes.

Our simulation study also made clear the different strength of the proposed method and SAM. When the sample-size was as small as 10, the absolute bias in SAM was smaller than that in the proposed method, while the variance was almost the same between them. This strength of SAM may be attractive because microarray experiments are often conducted with small sample-sizes. When the number of differentially expressed genes was as small as 10% of the total genes, FDR were more accurately estimated in SAM than the proposed method. An additional simulation experiment with no differentially expressed genes, i.e. *s =* 0, revealed that the bias and variance of estimated FDR in SAM were slightly smaller than that in the proposed method. When the sample-size or the number of differentially expressed genes was large, however, both the bias and variance in the proposed method were smaller than those in SAM, probably because SAM could not accurately estimate the distribution of non-differentially expressed genes. The proposed method has an advantage over SAM when the sample-size is greater than 20 or the number of differentially expressed genes is greater than 10% of the total genes. Thus, the proposed method outperforms SAM when the sample-size of each group is more than 20 or the proportion of differentially expressed genes is more than 10% irrespective of the target FDR. Otherwise, SAM outperforms the proposed method.

There would be many over-expressed genes in responder group relative to non-responder group based on both Figures 1–2 in the actual data, whereas under-expressed genes would be few. Table 4 shows the estimated FDR and the number of identified genes in both methods when the cutoff value is changed from 0.1 to 2.0 by 0.1. The number of identified genes was equal between the two methods, because the same *t*-type score and cut-off value was used. According to the result of simulation study, FDR by the proposed method may be slightly underestimated since the sample-size of the responder group and non-responder group were 10 and 14, respectively, in the actual data. However, the degree of underestimation would not be so large that its influence might be cancelled by taking a slightly smaller value of estimated FDR than the target FDR. For instance, the estimated FDR by the proposed method is 0.007, which corresponded to the cut-off value 1.7 in Table 4, may be appropriate for the target FDR 0.01. If so, 280 genes were identified as the differentially expressed genes correlated with the docetaxel response.

## Figures and Tables

**Table 1.** Results of simulation situation 1.

**Target FDR (*tFDR*)**	**Proposed method**		**SAM**	
	**Bias**	**Variance**	**Bias**	**Variance**
0.01	−0.0012	0.0057	0.0005	0.0071
0.05	−0.0044	0.0163	0.0019	0.0184
0.10	−0.0045	0.0214	0.0027	0.0247
0.20	−0.0055	0.0239	0.0035	0.0321
0.50	−0.0035	0.0154	0.0142	0.0397

**Table 2.** Results of simulation situation 2.

**Sample-size (*N*)**	**Proposed method**		**SAM**	
	**Bias**	**Variance**	**Bias**	**Variance**
5	−0.0308	0.0361	0.0005	0.0340
10	−0.0122	0.0257	0.0013	0.0259
20	−0.0045	0.0214	0.0027	0.0247
40	−0.0034	0.0198	0.0042	0.0260
80	−0.0032	0.0205	0.0085	0.0258

**Table 3.** Results of simulation situation 3

**Number of differentially expressed genes (*s*)**	**Proposed method**		**SAM**	
	**Bias**	**Variance**	**Bias**	**Variance**
30	−0.0094	0.0456	−0.0004	0.0549
75	−0.0072	0.0290	0.0025	0.0346
150	−0.0045	0.0214	0.0027	0.0247
300	−0.0032	0.0138	−0.0025	0.0176
600	−0.0022	0.0087	−0.0102	0.0129

**Table 4.** Results of application of the proposed method and SAM. The estimated FDR in both methods, and the number of identified genes for each cut-off value.

**Cut-off value**	**Estimated FDR**		**Number of identified genes**
	**Proposed method**	**SAM**	
0.1	0.504	0.748	6,433
0.2	0.464	0.612	5,685
0.3	0.420	0.487	4,935
0.4	0.373	0.381	4,227
0.5	0.324	0.291	3,612
0.6	0.274	0.225	3,008
0.7	0.226	0.172	2,506
0.8	0.181	0.132	2,063
0.9	0.141	0.101	1,680
1.0	0.106	0.076	1,371
1.1	0.078	0.059	1,087
1.2	0.056	0.044	877
1.3	0.039	0.033	691
1.4	0.026	0.024	571
1.5	0.017	0.018	451
1.6	0.011	0.014	357
1.7	0.007	0.011	280
1.8	0.004	0.008	218
1.9	0.003	0.006	171
2.0	0.002	0.006	119
